# Preconception Thyrotropin Levels and Risk of Adverse Pregnancy Outcomes in Chinese Women Aged 20 to 49 Years

**DOI:** 10.1001/jamanetworkopen.2021.5723

**Published:** 2021-04-13

**Authors:** Ying Yang, Tonglei Guo, Jinrong Fu, Jian Kuang, Yuanyuan Wang, Ya Zhang, Hongguang Zhang, Yuan He, Zuoqi Peng, Qiaomei Wang, Haiping Shen, Yiping Zhang, Donghai Yan, Xu Ma, Haixia Guan

**Affiliations:** 1 National Research Institute for Family Planning, Haidian District, Beijing, China; 2 National Human Genetic Resource Center, Haidian District, Beijing, China; 3 Graduate School of Peking Union Medical College, Dongdan Santiao, Dongcheng District, Beijing, China; 4 School of Public Health, Hebei Medical University, Changan District, Shijiazhuang, China; 5 Department of Endocrinology and Metabolism, The First Hospital of China Medical University, Shenyang, China; 6 Department of Endocrinology, Guangdong Provincial People's Hospital, Guangdong Academy of Medical Sciences, Guangzhou, China; 7 Department of Maternal and Child Health, National Health Commission of the People’s Republic of China, Xicheng District, Beijing, China

## Abstract

**Importance:**

Maternal thyrotropin levels during gestation have a profound effect on pregnancy outcomes; however, few studies to date have evaluated the importance of preconception thyrotropin levels.

**Objective:**

To investigate the associations between preconception thyrotropin levels and pregnancy outcomes.

**Design, Setting, and Participants:**

This population-based cohort study enrolled Chinese women aged 20 to 49 years from the National Free Prepregnancy Checkups Project in China. Participants conceived within 6 months after the thyrotropin examination and completed follow-up for pregnancy outcomes between January 1, 2013, and December 31, 2017. Data were analyzed between May 1, 2019, and March 31, 2020.

**Exposures:**

Levels of thyrotropin within 6 months before pregnancy, measured as less than 0.10 mIU/L, 0.10 to 0.36 mIU/L, 0.37 to 2.49 mIU/L, 2.50 to 4.87 mIU/L, 4.88 to 9.99 mIU/L, and 10.00 mIU/L or greater.

**Main Outcomes and Measures:**

The association of maternal preconception thyrotropin levels with the 4 primary adverse pregnancy outcomes was assessed, including preterm birth (PTB), small for gestational age (SGA), birth defect, and perinatal infant death. Logistic regression analyses were used to evaluate the association between preconception maternal thyrotropin levels and risk of adverse pregnancy outcomes. The dose-response associations were assessed using restricted cubic spline regression.

**Results:**

This study enrolled 5 840 894 women (mean [SD] age, 26.30 [4.10] years) in the primary analysis. The median (interquartile range [IQR]) thyrotropin level was 1.60 (1.06-2.37) mIU/L. The cumulative incidences for the adverse pregnancy outcomes were as follows: PTB, 6.56%; SGA, 7.21%; birth defect, 0.02%; and perinatal infant death, 0.33%. Compared with the reference group (thyrotropin range, 0.37-2.49 mIU/L), both low (<0.10 mIU/L and 0.10-0.36 mIU/L) and high (4.88-9.99 mIU/L and ≥10.00 mIU/L) maternal preconception thyrotropin levels were associated with higher risk of PTB (low: odds ratio [OR], 1.23 [95% CI, 1.19-1.27] and OR, 1.15 [95% CI, 1.13-1.18] vs high: OR, 1.13 [95% CI, 1.10-1.15] and OR, 1.14 [95% CI, 1.08-1.20]), SGA (low: OR, 1.37 [95% CI, 1.33-1.40] and OR, 1.14 [95% CI, 1.12-1.17] vs high: OR, 1.05 [95% CI, 1.03-1.08] and OR, 1.17 [95% CI, 1.11-1.23]), and perinatal infant death (low: OR, 1.26 [95% CI, 1.10-1.43] and OR, 1.14 [95% CI, 1.05-1.24] vs high: OR, 1.31 [95% CI, 1.20-1.43] and OR, 1.47 [95% CI, 1.21-1.80]). J-shaped associations between preconception thyrotropin levels and PTB (χ^2^ = 1033.45; nonlinear *P* < .001), SGA (χ^2^ = 568.90; nonlinear *P* < .001), and perinatal infant death (χ^2^ = 38.91; nonlinear *P* < .001) were identified.

**Conclusions and Relevance:**

In this cohort study, both low and high maternal thyrotropin levels were associated with a significantly increased risk of adverse pregnancy outcomes. Results suggest that the optimal preconception thyrotropin levels may be between 0.37 mIU/L and 2.50 mIU/L to prevent adverse pregnancy outcomes.

## Key Points

**Question:**

Are abnormal preconception thyrotropin levels associated with adverse maternal and fetal outcomes?

**Findings:**

In this populational-based cohort study of over 5.8 million Chinese women, both low and high maternal preconception thyrotropin levels were associated with higher risk of preterm birth, small for gestational age, and perinatal infant death.

**Meaning:**

In this study, both low and high maternal thyrotropin levels were associated with a significantly increased risk of adverse pregnancy outcomes; the optimal preconception thyrotropin levels may be between 0.37 mIU/L and 2.50 mIU/L.

## Teaser

This cohort study investigates whether abnormal preconception thyrotropin levels are associated with adverse maternal and fetal outcomes among Chinese women.

## Introduction

Normal maternal thyroid function is essential for achieving optimal pregnancy outcomes, especially during the early gestation period.^1^ Unfortunately, maternal thyroid dysfunction is relatively common during pregnancy, with an overall prevalence of 0.61% for overt hypothyroidism, 5.1% for subclinical hypothyroidism, 0.64% for overt hyperthyroidism, and 1.77% for subclinical hyperthyroidism.^2^

Maternal thyroid dysfunction during pregnancy increases the risk of multiple adverse fetomaternal outcomes, and early intervention may be effective to reduce this risk. In a recent study, Jansen et al^3^ found that abnormal maternal thyroid function was associated with lower total gray matter volume and cortical gray matter volume in offspring, but this association was no longer evident after approximately 14 weeks of gestation. Furthermore, treatment of thyroid dysfunction during pregnancy may be more effective during early gestational weeks, with little to no benefit in late gestational weeks. Two major studies from the UK and the US demonstrated that initiating treatment for maternal subclinical hypothyroidism at 12 weeks of gestation or later did not improve cognitive outcomes in children.^4,5^ However, results from the Tehran Thyroid and Pregnancy Study indicated that in women with subclinical hypothyroidism and negative anti–thyroid peroxidase antibodies, levothyroxine treatment initiated during the first trimester could lower the relative risk of preterm birth (PTB) by more than 60%.^6^ These findings revealed the time-dependent role of maternal thyroid hormones, suggesting an early and narrow window for effective diagnosis and intervention to improve maternal and fetal health.

Although screening for thyroid dysfunction during pregnancy is recommended by medical guidelines,^7,8^ there are, to our knowledge, limited studies on preconception screening. Furthermore, controversies remain on whether to perform universal screening or targeted screening. Based on current evidence, the optimal time for screening is believed to be earlier rather than later. Earlier screening allows for treatment initiation within the window for effective intervention to correct abnormal thyroid function and to mitigate the risk for adverse fetal development owing to maternal thyroid dysfunction. In addition, some researchers have suggested screening for preconception thyroid dysfunction for prompt and appropriate management. However, most previous studies focused on the effect of maternal thyroid function during pregnancy, and there are limited data on the association of preconception thyroid function with obstetric outcomes and on whether thyroid function reference values for the general population and pregnant women are suitable for women before conception.

Prompted by considerable ambiguities about preconception thyroid function management, we conducted this large-scale, population-based cohort study to elucidate the association between maternal preconception thyrotropin levels and various adverse pregnancy outcomes and to evaluate the optimal thyrotropin range in women planning for pregnancy.

## Methods

### Data Sources and Study Population

**Figure 1.**
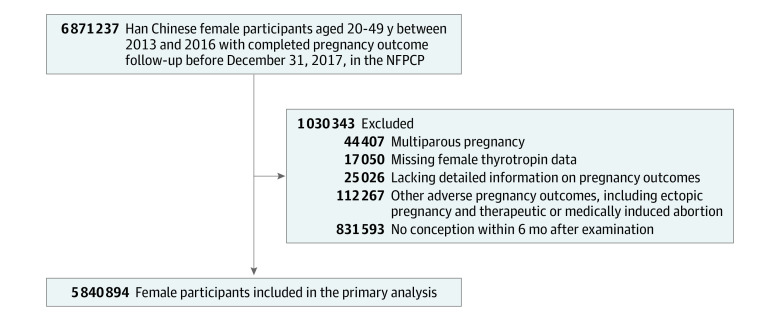
Flowchart of the Study Population NFPCP indicates National Free Prepregnancy Checkups Project.

All participants of this cohort study were from the National Free Prepregnancy Checkups Project (NFPCP) cohort. The NFPCP is a national preconception health care service supported by the National Health Commission and the Ministry of Finance of the People’s Republic of China. It aims to provide free preconception health examinations and counseling for rural and urban reproductive-aged couples who plan to conceive within the next 6 months. Detailed information of the design, organization, and implementation of the NFPCP has been described previously.^9,10,11^ Between January 1, 2013, and December 31, 2016, a total of 6 871 237 Han Chinese women aged 20 to 49 years from 2679 counties in 31 provinces participated in the NFPCP and successfully became pregnant after the preconception examination. All of them completed follow-up for their pregnancy outcome by December 31, 2017. Those women who had a multiparous pregnancy (n = 44 407) were excluded. Women who were missing information on preconception thyrotropin data (n = 17 050), did not have detailed records of pregnancy outcomes (n = 25 026), or experienced certain pregnancy outcomes, including ectopic pregnancy or medically induced abortion (n = 112 267), were excluded. Among the remaining 6 672 487 women, 5 840 894 women successfully conceived within 6 months after the NFPCP preconception examination, whereas 831 593 did not conceive until more than 6 months after the preconception examination. Because the preconception thyrotropin data are further from the date of conception in the latter group, these women were excluded from the current study (Figure 1). Data were analyzed between May 1, 2019, and March 31, 2020.

This study was approved by the institutional research review board at the National Research Institute for Family Planning in Beijing, China. Written informed consent was obtained from all NFPCP participants. This study followed the Strengthening the Reporting of Observational Studies in Epidemiology (STROBE) reporting guideline.

### Measurements and Pregnancy Outcomes

For each individual who participated in the NFPCP, different types of data were collected and archived in 3 main stages: preconception health examinations, early pregnancy follow-up, and pregnancy outcome follow-up (eAppendix in the Supplement).

Four adverse pregnancy outcomes were studied. (1) PTB was defined as live birth between 28 and 36 completed weeks of pregnancy; (2) a small for gestational age (SGA) infant was defined by a newborn birth weight by gestational age and sex below the 10th percentile; (3) birth defects were defined as structural, functional, or metabolic abnormalities in the fetus occurring before birth, including cleft lip, anencephaly, cerebrospinal meningitis, hydrocephalus, open spina bifida, cleft palate, trisomy 21 syndrome, and congenital heart disease; and (4) perinatal infant death was defined as stillbirth after 28 weeks of gestation or newborns who died within 7 days after birth. We assessed associations of maternal preconception thyrotropin levels with each of these 4 primary adverse pregnancy outcomes.

### Serum Thyrotropin Measurements, Calculations of Thyrotropin Reference Range, and Thyrotropin Multiples of the Median Value

During the preconception examination, blood samples after at least 8 hours of fasting were collected from each participant and immediately sent to the laboratories of local maternal and child service centers. Serum thyrotropin levels were measured in accordance with National Guide to Clinical Laboratory Procedures. Because of the huge number of participants in this national survey, it was impractical to adopt a uniform thyrotropin detection kit or even detection method across all local laboratories. Each county consistently used a single type of thyrotropin assay, either enzyme-linked immunosorbent assay kits or electrochemiluminescence immunoassay.

In this study, a population-specific reference range of thyrotropin was established for further analysis. The reference population was defined as Han Chinese women who gave birth to a healthy infant and did not have any of the following conditions: (1) history of adverse pregnancy outcomes, thyroid disorders, diabetes, hypertension, or anemia; (2) currently using medications; (3) exposure to cigarettes or alcohol; and (4) abnormal body mass index (BMI) at baseline. This reference population consisted of 1 646 039 women. The 2.5th, 50th (median), and 97.5th percentiles for thyrotropin levels were 0.37 mIU/L, 1.66 mIU/L, and 4.88 mIU/L, respectively.

To overcome the variability or systematic differences between various laboratories or assays in addition to the absolute value of thyrotropin measurement, preconception thyrotropin levels were converted to multiples of the median (MoM) for some analyses. A thyrotropin MoM value was calculated by dividing the individual’s thyrotropin level by the median thyrotropin level in the reference population of the county where she registered to do the preconception examination.

### Statistical Analysis

Continuous variables with normal distribution were expressed as mean values (SDs), and nonnormally distributed variables were expressed as medians (interquartile ranges [IQRs]). Categorical variables were expressed as numbers (percentages) for baseline characteristics according to different preconception thyrotropin levels. The χ^2^ test, Mann-Whitney *U* test, or Kruskal-Wallis test was used to compare the distributions of baseline characteristics among the different groups.

The age-adjusted and multivariable-adjusted odds ratios (ORs) were estimated by logistic regression models to examine the association between preconception thyrotropin levels and thyrotropin MoM values with the risk of adverse pregnancy outcomes. According to preconception thyrotropin levels, the women were divided into 6 groups in all the logistic regressions: group 1, less than 0.10 mIU/L; group 2, 0.10 to 0.36 mIU/L; group 3, 0.37 to 2.49 mIU/L; group 4, 2.50 to 4.87 mIU/L; group 5, 4.88 to 9.99 mIU/L; and group 6, greater than or equal to 10.00 mIU/L. The reference group consisted of women with a thyrotropin level of 0.37 to 2.49 mIU/L.

The potential confounding variables, which were significant in univariate analyses, were subsequently adjusted in multivariable models. These variables included maternal age at last menstrual period (categorized as age 20-24.9 years, 25-29.9 years, 30-34.9 years, 35-39.9 years, and ≥40 years); higher education (senior high school, college, or higher); preconception BMI (<18.5 kg/m^2^, 18.5-23.9 kg/m^2^, 24.0-27.9 kg/m^2^, and ≥28.0 kg/m^2^); area of residence (rural or urban); alcohol drinking; passive smoking; history of thyroid disease, diabetes, or hypertension; and history of adverse pregnancy outcomes.

Sensitivity analyses were conducted by excluding participants with missing data on baseline characteristics. The dose-response relationship of maternal preconception thyrotropin levels or thyrotropin MoM values and risk of adverse pregnancy outcomes were assessed using restricted cubic spline (RCS) regression, and 4 knots at the 5th, 35th, 65th, and 95th percentiles of preconception thyrotropin levels were used in plotted smooth curves. *P* values for linear correlation (multivariable linear regression) or nonlinear correlation (generalized linear regression) were used to examine the trend of curves between maternal preconception thyrotropin levels or thyrotropin MoM values and risk of adverse pregnancy outcomes; covariates were the same as the logistic regression.

Statistical analysis was performed using R software, version 3.5.0 (R Foundation for Statistical Computing) with the analysis packages epade, version 0.3.8; forestplot, version 1.7.2; rms, version 5.1-2; ggplot2, version 3.1.0; reshape2, version 1.4.3; and speedglm, version 0.3-2. All statistical tests were 2-sided, and *P* < .05 was considered statistically significant.

## Results

Among 6 871 237 women who completed follow-up for their pregnancy outcomes from 2013 to 2017 in the NFPCP, 1 030 343 participants were excluded. A total of 5 840 894 women (mean [SD] age, 26.30 [4.10] years) were eventually included in the primary analysis (Figure 1). Demographic characteristics of the women are presented in the Table.

**Table.** Characteristics of Female Participants According to Preconception Thyrotropin Levels^a^

Characteristic	Thyrotropin, median (IQR), mIU/L (n = 5 840 894)	Maternal preconception thyrotropin levels, mIU/L			
		<0.37 (n = 223 181)	0.37-4.87 (n = 5 470 626)	≥4.88 (n = 147 087)	*P* value^b^
Thyrotropin, median (IQR), mIU/L	1.60 (1.31)	0.21 (0.21)	1.61 (1.24)	5.94 (2.38)	NA
Age at LMP, y						
20-24.9	1.58 (1.32)	100 149 (44.87)	2 360 638 (43.15)	58 413 (39.71)	<.001^c^
25-29.9	1.60 (1.30)	88 980 (39.87)	2 238 871 (40.93)	61 183 (41.60)	
30-34.9	1.63 (1.32)	25 220 (11.30)	634 559 (11.60)	19 377 (13.17)	
35-39.9	1.67 (1.35)	7547 (3.38)	199 074 (3.64)	6743 (4.58)	
≥40	1.68 (1.36)	1281 (0.57)	37 323 (0.68)	1365 (0.93)	
Parity						
0	1.59 (1.32)	150 133 (67.27)	3 553 031 (64.95)	90 602 (61.60)	<.001
≥1	1.62 (1.32)	72 906 (32.67)	1 912 884 (34.97)	56 337 (38.30)	
Missing data	NA	136 (0.06)	4544 (0.08)	136 (0.09)	
Education						
High school or above	1.62 (1.33)	46 356 (20.77)	1 058 064 (19.34)	30 803 (20.94)	<.001
Primary school or below	1.60 (1.31)	175 514 (78.64)	4 391 532 (80.27)	115 740 (78.69)	
Missing data	NA	1331 (0.59)	21 030 (0.38)	544 (0.37)	
Residence						
Rural	1.59 (1.31)	201 412 (90.25)	4 973 699 (90.92)	130 494 (88.72)	<.001
Urban	1.67 (1.37)	21 767 (9.75)	496 809 (9.08)	16 590 (11.28)	
Missing data	NA	2 (<0.001)	118 (<0.001)	3 (<0.001)	
BMI						
Underweight (<18.5)	1.56 (1.34)	43 317 (19.41)	761 952 (13.93)	20 244 (13.76)	<.001
Normal weight (18.5-23.9)	1.60 (1.30)	151 541 (67.90)	3 880 983 (70.94)	100 679 (68.45)	
Overweight (24.0-27.9)	1.62 (1.33)	23 085 (10.34)	668 600 (12.22)	20 502 (13.94)	
Obesity (≥28.0)	1.67 (1.39)	4894 (2.19)	152 918 (2.80)	5470 (3.72)	
Missing data	NA	344 (0.15)	6173 (0.11)	192 (0.13)	
Alcohol consumption						
Yes	1.69 (1.40)	6158 (2.76)	144 217 (2.64)	4952 (3.37)	<.001
No	1.60 (1.31)	216 727 (97.11)	5 318 970 (97.23)	141 953 (96.51)	
Missing data	NA	296 (0.13)	7439 (0.14)	182 (0.12)	
Secondhand smoke						
Yes	1.72 (1.46)	28 592 (12.81)	618 241 (11.30)	20 769 (14.12)	<.001
No	1.59 (1.31)	194 253 (87.04)	4 845 767 (88.58)	126 124 (85.75)	
Missing data	NA	336 (0.15)	6618 (0.12)	194 (0.13)	
Hypertension						
Yes	1.74 (1.45)	3436 (1.54)	83 584 (1.53)	3500 (2.38)	<.001
No	1.60 (1.31)	219 626 (98.41)	5 382 942 (98.40)	143 471 (97.54)	
Missing data	NA	119 (0.05)	4100 (0.07)	116 (0.08)	
Diabetes					
Yes	1.41 (1.34)	4951 (2.22)	61 184 (1.12)	1835 (1.25)	<.001
No	1.60 (1.31)	218 110 (97.73)	5 405 270 (98.81)	145 128 (98.67)	
Missing data	NA	120 (0.05)	4172 (0.07)	124 (0.08)	
History of adverse pregnancy outcomes						
Yes	1.62 (1.36)	57 827 (25.91)	1 281 488 (23.42)	39 956 (27.16)	<.001
No	1.60 (1.30)	164 077 (73.52)	4 156 610 (75.98)	106 171 (72.18)	
Missing data	NA	1277 (0.57)	32 528 (0.59)	960 (0.65)	

Abbreviations: BMI, body mass index (calculated as weight in kilograms divided by height in meters squared); IQR, interquartile range; LMP, last menstrual period; NA, not available.

^a^ Data presented as number (percentage) unless otherwise indicated.

^b^ Multiple comparison with Bonferroni-adjusted *P* < .05 compared with the thyrotropin 0.37-4.87 mIU/L group (<0.37 mIU/L group vs 0.37-4.87 mIU/L group; ≥4.88 mIU/L group vs 0.37-4.87 mIU/L group).

^c^ The Kruskal-Wallis H test was used to examine the differences of baseline characteristics among thyrotropin groups; otherwise, the χ^2^ test was used.

### Preconception Levels of Serum Thyrotropin 

The median thyrotropin level was 1.60 (IQR, 1.06-2.37) mIU/L among 5 840 894 women who had successfully conceived within 6 months after the preconception examination. This level is significantly lower than that among the women who conceived later than 6 months (n = 831 593; median, 1.76 [IQR, 1.19-2.56] mIU/L) (eTable 1 in the Supplement). Among the 5 840 894 included women, 223 181 (3.82%) had a subnormal thyrotropin level (<0.37 mIU/L), and 147 087 (2.52%) had a supranormal thyrotropin level (≥4.88 mIU/L). The number of women with thyrotropin levels within the reference range (0.37-4.87 mIU/L) was 5 470 626 (93.66%), including 4 310 340 women (73.80%) with thyrotropin levels between 0.37 and 2.49 mIU/L and 1 160 286 (19.86%) with thyrotropin levels between 2.50 and 4.87 mIU/L.

Compared with women who had a thyrotropin level between 0.37 and 4.87 mIU/L, those who had abnormal thyrotropin levels (≥4.88 or <0.37 mIU/L) were more likely to have been exposed to secondhand smoking (20 769 [14.12%] or 28 592 [12.81%] vs 618 241 [11.30%]), to consume alcohol (4952 [3.37%] or 6158 [2.76%] vs 144 217 [2.64%]), to have higher educational attainment (30 803 [20.94%] or 46 356 [20.77%] vs 1 058 064 [19.34%]), or to have a history of adverse pregnancy outcomes (39 956 [27.16%] or 57 827 [25.91%] vs 1 281 488 [23.42%]). Women with thyrotropin levels of at least 4.88 mIU/L were also more likely to be older (≥40 years, 1365 [0.93%] vs 1281 [0.57%] or 37 232 [0.68%]) and have a higher BMI (>28 kg/m^2^, 5470 [3.72%] vs 4894 [2.19%] or 152 918 [2.80%]) and a higher blood pressure level (hypertension, 3500 [2.38%] vs 3436 [1.54%] or 83 584 [1.53%]) than women with thyrotropin levels less than 0.37 mIU/L or between 0.37 and 4.87 mIU/L, respectively. Meanwhile, women with thyrotropin levels less than 0.37 mIU/L were more likely to be underweight (43 317 [19.41%] vs 761 952 [13.93%] or 20 244 [13.76%]) and have preexisting diabetes (4951 [2.22%] vs 61 184 [1.12%] or 1835 [1.25%]) than women with a thyrotropin level between 0.37 and 4.87 mIU/L or at least 4.88 mIU/L, respectively (Table).

### Risk of Adverse Pregnancy Outcomes According to Thyrotropin Groups

The median time to pregnancy since baseline examination was 1.56 (IQR, 0.49-3.23) months. The cumulative incidence for each of the adverse pregnancy outcomes were as follows: PTB, 6.56%; SGA, 7.21%; birth defect, 0.02%; and perinatal infant death, 0.33%. We evaluated associations of maternal preconception thyrotropin level with the 4 primary adverse pregnancy outcomes.

**Figure 2.**
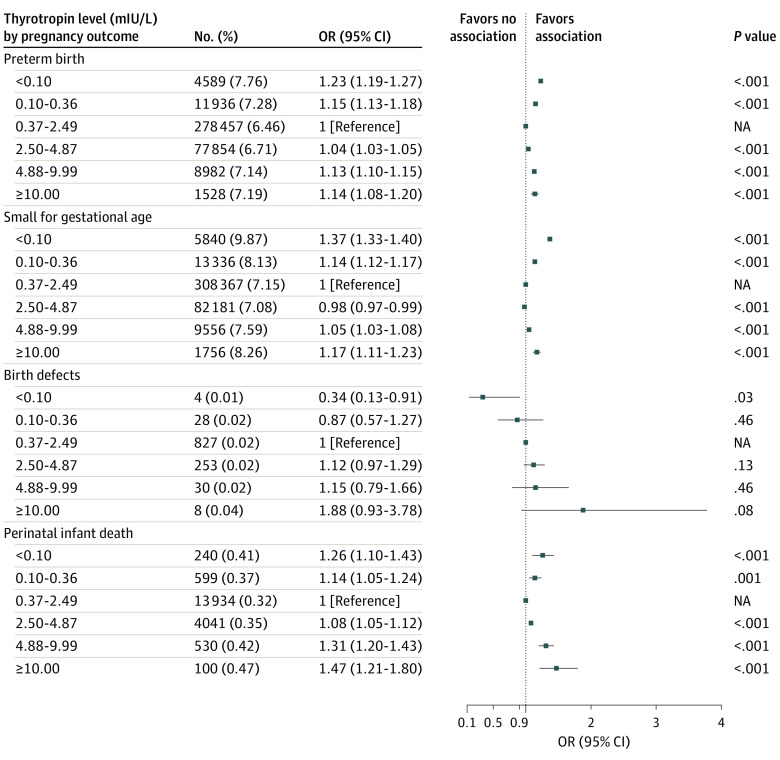
Adjusted Odds Ratios (ORs) of Adverse Pregnancy Outcomes According to Preconception Thyrotropin Levels All multivariable-adjusted ORs were estimated by logistic regression models and adjusted by maternal age at the last menstrual period, maternal education, area of residence, preconception body mass index, alcohol drinking, passive smoking, history of thyroid disease, hypertension, diabetes, and history of adverse pregnancy outcomes. NA indicates not available.

Compared with the reference group (thyrotropin, 0.37-2.49 mIU/L), both low (<0.10 mIU/L and 0.10-0.36 mIU/L) and high (4.88-9.99 mIU/L and ≥10.00 mIU/L) maternal preconception thyrotropin levels were associated with higher risk of PTB (low: OR, 1.23 [95% CI, 1.19-1.27] and OR, 1.15 [95% CI, 1.13-1.18] vs high: OR, 1.13 [95% CI, 1.10-1.15] and OR, 1.14 [95% CI, 1.08-1.20]), SGA (low: OR, 1.37 [95% CI, 1.33-1.40] and OR, 1.14 [95% CI, 1.12-1.17] vs high: OR, 1.05 [95% CI, 1.03-1.08] and OR, 1.17 [95% CI, 1.11-1.23]), and perinatal infant death (low: OR, 1.26 [95% CI, 1.10-1.43] and OR, 1.14 [95% CI, 1.05-1.24] vs high: OR, 1.31 [95% CI, 1.20-1.43] and OR, 1.47 [95% CI, 1.21-1.80]). Detailed multivariable-adjusted ORs (95% CIs) are listed in Figure 2 and eTable 2 in the Supplement. Similar results were observed in sensitivity analysis after excluding participants with missing data on baseline characteristics (eTable 3 in the Supplement). Similar results were observed when using 0.37 to 4.87 mIU/L of thyrotropin as the reference group (eTable 4 in the Supplement).

### Dose-Response Association Between Maternal Preconception Thyrotropin or Thyrotropin MoM Values and Various Adverse Pregnancy Outcomes

**Figure 3.**
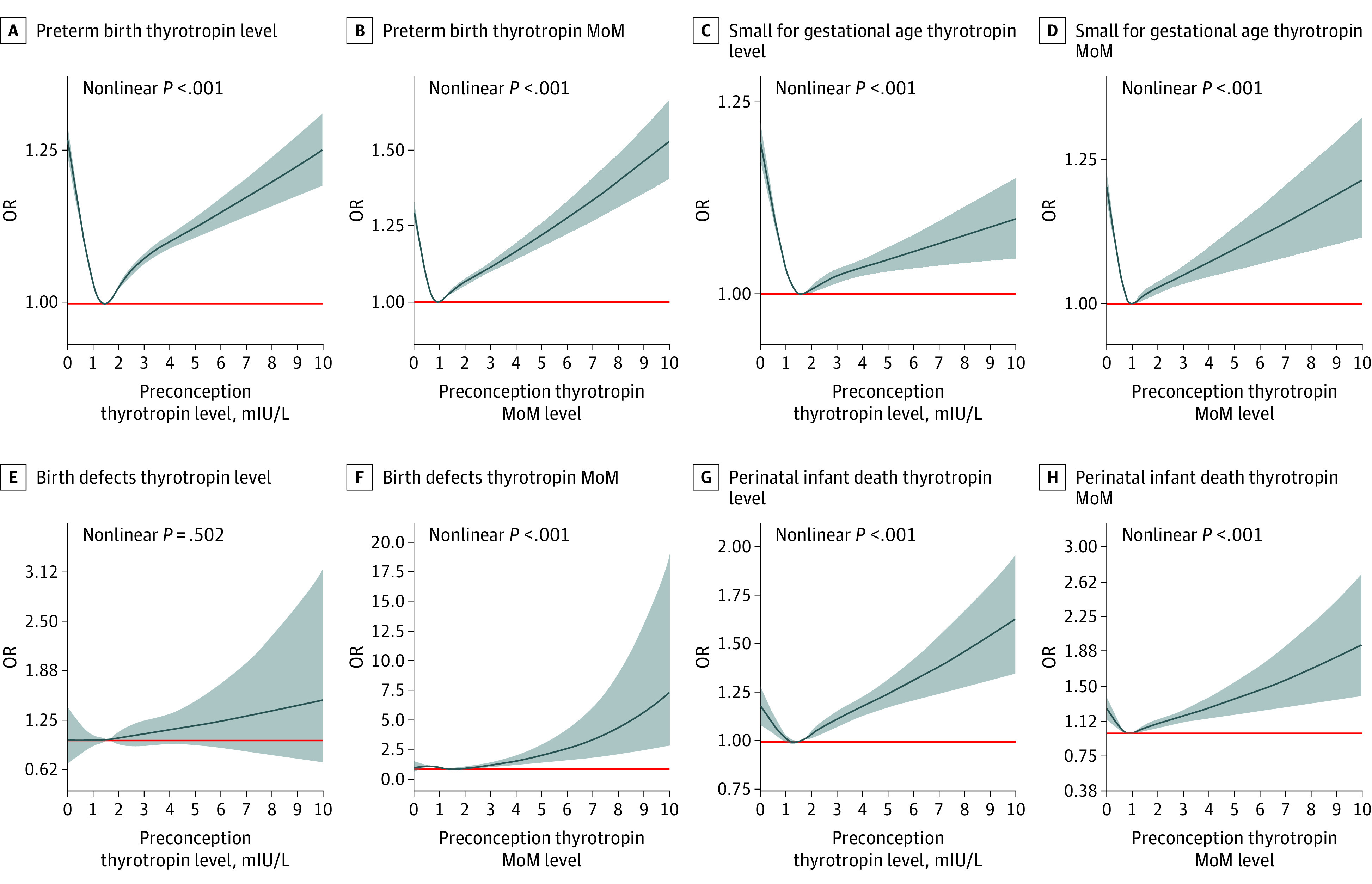
Dose-Response Relationship Between Maternal Preconception Thyrotropin or Thyrotropin Multiples of the Median (MoM) and the Risk of Adverse Pregnancy Outcomes The multivariable-adjusted odds ratios (ORs) are shown for the associations between maternal preconception thyrotropin levels or thyrotropin MoM values and the risk of preterm birth (A, B), small for gestational age (C, D), birth defects (E, F), and perinatal infant death (G, H). Dark blue curves and shaded gray areas show predicted ORs and 95% CIs, respectively. Maternal age at last menstrual period, higher education, area of residence, body mass index, alcohol drinking, passive smoking, history of thyroid disease, hypertension, diabetes, and history of adverse pregnancy outcomes were used in the analysis as covariates. The red lines represent the reference level. (A: thyrotropin = 1.50 mIU/L, OR = 1; B: thyrotropin MoM = 0.90, OR = 1; C: thyrotropin = 1.65 mIU/L, OR = 1; D: thyrotropin MoM = 0.95, OR = 1; E: thyrotropin = 0.90 mIU/L, OR = 1; F: thyrotropin MoM = 1.45, OR = 1; G: thyrotropin = 1.36 mIU/L, OR = 1; and H: thyrotropin MoM = 0.90, OR = 1).

The RCS results revealed a J-shaped dose-response association of maternal preconception thyrotropin or thyrotropin MoM levels with PTB (χ^2^ = 1033.45; nonlinear *P* < .001 or χ^2^ = 1025.75; nonlinear *P* < .001, respectively) (Figure 3A and B), SGA (χ^2^ = 568.90; nonlinear *P* < .001 or χ^2^ = 528.91; nonlinear *P* < .001, respectively) (Figure 3C and D), and perinatal infant death (χ^2^ = 38.91; nonlinear *P* < .001 or χ^2^ = 465.35; nonlinear *P* < .001, respectively) (Figure 3G and H). There was no statistically significant association between preconception thyrotropin and birth defects (χ^2^ = 0.03; nonlinear *P* = .50) (Figure 3E), whereas increased preconception thyrotropin MoM was positively associated with the risk of birth defects (χ^2^ = 10.23; nonlinear *P* < .001) (Figure 3F).

## Discussion

In this large, population-based, retrospective cohort study of more than 5.8 million Chinese women, we observed that abnormal thyrotropin levels within 6 months before pregnancy were associated with adverse pregnancy outcomes. The risk of PTB, SGA, and perinatal infant death showed a J-shaped association curve with the increase in preconception thyrotropin levels. A preconception thyrotropin within 0.37 and 2.49 mIU/L was associated with the lowest risk of adverse pregnancy outcomes. Our findings enhance our knowledge underpinning thyroid disease and reproductive health.

To our knowledge, limited evidence existed before the present study in terms of the association of slightly decreased thyrotropin levels with pregnancy outcomes. Casey et al^12^ reported that women with subclinical hyperthyroidism had a lower risk of hypertension and that the prevalence of other adverse pregnancy outcomes was comparable with that of women who were euthyroid. However, our findings suggest that thyrotoxicosis before or during early pregnancy should not be ignored. In addition, overt hyperthyroidism during pregnancy has been reported to increase the risk of adverse pregnancy outcomes, including miscarriage, PTB, and neonatal death.^12,13,14,15,16,17^ The exposure to excessive thyroid hormones may also affect child brain morphology and elevate the risk of mental disorders in offspring.^3,18^ Therefore, appropriate management for thyrotoxicosis, in particular overt hyperthyroidism, should be provided before pregnancy.

The adverse effects of hypothyroidism during pregnancy were affirmed in previous studies,^3,19,20,21^ and treatment with levothyroxine showed beneficial effects.^6,22,23^ Medical guidelines are in agreement that hypothyroidism should be detected and managed as early as possible in pregnant women. Of note, when serum thyrotropin levels are within the reference range but higher than 2.50 mIU/L, there might be elevated risk of pregnancy loss,^24,25,26^ gestational diabetes, and low birth weight.^27^ Although fetal development in terms of birth weight was not associated with normal range maternal thyrotropin levels during early pregnancy,^28,29^ whether high-normal thyrotropin levels have clinical relevance remains controversial. Regardless, medical guidelines recommend a thyrotropin target of approximately 2.50 mIU/L for levothyroxine treatment of hypothyroidism during pregnancy. Our study results suggest that preconception hypothyroidism was associated with elevated risk of adverse pregnancy outcomes. Study results also revealed that a preconception thyrotropin level between the lower reference limit of 0.37 mIU/L and 2.50 mIU/L may be optimal, as this range was associated with the lowest risk of adverse pregnancy outcomes. In consideration of this study’s results, we propose that advancing the management time window to the preconception period might prevent the occurrence and exacerbation of hypothyroidism during pregnancy. Furthermore, maintaining preconception thyrotropin levels within the low-normal range (approximately 2.50 mIU/L) might be the most beneficial approach.

In contrast with a study with a small sample size (n = 78) conducted by Khan and colleagues,^30^ which reported that preconception thyrotropin greater than or equal to 2.50 mIU/L was not associated with increased risk of adverse pregnancy outcomes, our study identified a significant association between preconception thyrotropin levels and the increased risk of various adverse pregnancy outcomes, even when thyrotropin levels were greater than 2.50 mIU/L but within the normal range. A previous study within the NFPCP cohort from 2010 to 2012 also reported an increased risk of PTB among women with elevated preconception thyrotropin levels.^31^ Based on these results, obstetricians and endocrinologists should be vigilant about the potential hazard of supranormal thyrotropin and high-normal thyrotropin levels for women of reproductive age. These findings also support the current guideline recommendations that levothyroxine dose should be adjusted to achieve a thyrotropin level between the lower reference limit and 2.50 mIU/L in women with hypothyroidism who are planning for pregnancy.^7,8^

Information on medical treatment for thyroid dysfunction during pregnancy was not collected and recorded in the NFPCP database. Nonetheless, based on the significantly higher risk of adverse pregnancy outcomes in women with preconception hypothyroidism, we speculate that hypothyroidism was not well managed in this cohort. We also noticed that the risk of any type of adverse pregnancy outcomes increased continuously among women with thyrotropin levels ranging between 4 and 10 mIU/L but attenuated among women with thyrotropin levels of at least 10 mIU/L. Possible explanations for these phenomena include the following: (1) participants of the current study came from 2679 counties across China, which may offer varying levels of medical service; (2) patients and primary care doctors may have lacked knowledge on how to address hypothyroidism during the periconceptional period; and (3) compared with a thyrotropin level of at least 10 mIU/L, which was a more obvious condition requiring treatment,^7^ mild hypothyroidism in women was less likely to receive appropriate intervention. These results emphasize the importance of continuing education for medical professionals and the public and the necessity for increased awareness about subclinical hypothyroidism in childbearing women. As an initial step, the Chinese government has put notable effort into the preconception thyroid screening program. The effectiveness and value of such a program would be impaired if the patients with hypothyroidism identified through screening were not properly treated.

### Strengths and Limitations

Compared with previous studies, this study has several strengths. First, this study analyzed the association between the preconception thyrotropin levels and the risk of adverse pregnancy outcomes based on the largest population-based cohort to date of more than 5.8 million participants. All serum thyrotropin measurements were collected and performed within 6 months before pregnancy, which may better represent a preconception thyroid function status. Second, in addition to thyrotropin levels, we used thyrotropin MoM values to calculate ORs and RCSs to enhance the accuracy of the results by eliminating the interference caused by various diagnostic assays. The characteristic of the thyrotropin MoM analysis also makes our findings more applicable to ordinary clinical practice in which different thyrotropin assay kits are used.

However, some limitations of the study should be mentioned. First, thyroid autoantibodies were not tested in the participants. With a prevalence of up to 18% in pregnant women, positive thyroid autoantibodies are associated with a higher risk of premature delivery.^32^ Nonetheless, Benhadi and colleagues^33^ found an association between high thyrotropin levels and spontaneous abortion and fetal or neonatal death after adjustment for thyroid peroxidase antibody status, suggesting that the influence of maternal thyrotropin levels on risk of adverse pregnancy outcomes cannot be obscured by thyroid autoimmunity. Second, we did not collect information about the thyroid-related medications used and longitudinal thyrotropin measurements. Therefore, classification solely according to preconception thyrotropin may have been inadequate to accurately reflect the association between serum thyrotropin levels and adverse pregnancy outcomes considering the dynamic thyrotropin alterations during pregnancy.^7^ We also did not follow up the intellectual and cognitive function of the offspring; neurodevelopment is a critical end point in fetal outcomes. However, it might be impracticable and cost-intensive to routinely follow up with such a large cohort. Third, even though previous studies reported that iodine deficiency was relevant to increased risk of PTB and low birth weight,^34^ we did not stratify the participants according to their iodine status because the relevant data were lacking. Nonetheless, China has implemented the universal salt iodization policy, and a recent national survey indicates that the majority of areas are iodine sufficient.^35^ Moreover, even though we have adjusted systemic errors by adapting MoM values and excluded confounding factors, there were still some unmeasured factors, including the use of assisted reproductive technology and pregnancy-related complications.

## Conclusions

In this cohort study, abnormal preconception thyrotropin levels were associated with increased risk of adverse pregnancy outcomes. From these results, we estimate that the optimal preconception thyrotropin level may be between the lower reference limit and 2.50 mIU/L. Our results suggest that advancing the thyrotropin screening time frame to the preconception period may be beneficial; however, more evidence and future analyses are needed.

## Notes

Supplement. **eAppendix.** Supplemental Methods **eTable 1.** The Differences of TSH Levels of Female Participants According to Time to Pregnancy **eTable 2.** Adjusted ORs of Adverse Pregnancy Outcomes According to Preconception TSH Levels **eTable 3.** Analysis of Association Between Preconception TSH and Adverse Pregnancy Outcomes After Excluding Participants With Missing Data on Baseline Characteristics (N = 5 765 420) **eTable 4.** Adjusted ORs of Adverse Pregnancy Outcomes According to Preconception TSH Levels Using 0.37-4.87 mIU/L of TSH as the Reference Group (N = 5 847 894)
