# Supplementary material for: Preconception Thyrotropin Levels and Risk of Adverse Pregnancy Outcomes in Chinese Women Aged 20 to 49 Years
Source: JAMA Netw Open. 2021 Apr 13;4(4):e215723. doi: 10.1001/jamanetworkopen.2021.5723 (PMC8044736; doi:10.1001/jamanetworkopen.2021.5723)
Supplement: Supplement. — eAppendix. Supplemental Methods eTable 1. The Differences of TSH Levels of Female Participants According to Time to Pregnancy eTable 2. Adjusted ORs of Adverse Pregnancy Outcomes According to Preconception TSH Levels eTable 3. Analysis of Association Between Preconception TSH and Adverse Pregnancy Outcomes After Excluding Participants With Missing Data on Baseline Characteristics (N = 5 765 420) eTable 4. Adjusted ORs of Adverse Pregnancy Outcomes According to Preconception TSH Levels Using 0.37-4.87 mIU/L of TSH as the Reference Group (N = 5 847 894) [file jamanetwopen-e215723-s001.pdf]

Supplementary Online Content

Yang Y, Guo T, Fu J, et al. Preconception thyrotropin levels and risk of adverse pregnancy outcomes in Chinese women aged 20 to 49 years. *JAMA Netw Open*. 2021;4(4):e215723. doi:10.1001/jamanetworkopen.2021.5723

eAppendix. Supplemental Methods

eTable 1. The Differences of TSH Levels of Female Participants According to Time to Pregnancy

eTable 2. Adjusted ORs of Adverse Pregnancy Outcomes According to Preconception TSH Levels

eTable 3. Analysis of Association Between Preconception TSH and Adverse Pregnancy Outcomes After Excluding Participants With Missing Data on Baseline Characteristics (N = 5 765 420)

eTable 4. Adjusted ORs of Adverse Pregnancy Outcomes According to Preconception TSH Levels Using 0.37-4.87 mIU/L of TSH as the Reference Group (N = 5 847 894)

This supplementary material has been provided by the authors to give readers additional information about their work.

© 2021 Yang Y et al. *JAMA Network Open.*

eAppendix. Supplemental Methods

In the first stage, preconception blood samples were collected at local maternal and child service centers to measure TSH. In addition, participant's demographics, past medical history, family history, dietary nutrition, lifestyle, environmental poisons, social-psychological factors, and physical examination were recorded in the preconception medical chart.

In the second stage, a telephone interview was conducted by trained staff to confirm the conception status of eligible participants. The first interview was conducted within 3 months after the completion of the first stage and was repeated every three months up to one year after the first stage until the participant self-reported as pregnant. Women who self-reported as pregnant were required to have their pregnancies confirmed by doctors and to undergo ultrasound imaging. The latter was used to calculate the first day of the last menstrual period (LMP). The ultrasound-adjusted LMP and information on toxic or harmful substances exposure, and any lifestyle changes during the first trimester of pregnancy were collected in this stage.

In the third stage, a pregnancy outcome follow-up telephone interview was performed by trained staff within one year after the LMP. Self-reported outcomes including ectopic pregnancy, medically-induced abortion, spontaneous abortion, induced labour, stillbirth, birth defects, preterm birth (PTB), and others. In addition, information regarding the delivery, such as delivery date, delivery mode, and birth weight, were collected. The gestational week was calculated by the duration between the ultrasound-adjusted LMP and the date of delivery.

© 2021 Yang Y et al. *JAMA Network Open.*

eTable 1. The Differences of TSH Levels of Female Participants According to Time to Pregnancy

	Pregnant within six months		Pregnant after six months		
	**n (%)**	**median (IQR)**	**n (%)**	**median (IQR)**	**P[a]**
	5840894 (38.96)	1.60 (1.31)	831593 (5.54)	1.76 (1.37)	<0.001
**TSH <0.37 mIU/L**	223181 (3.82)	0.21 (0.21)	18610 (2.24)	0.22 (0.25)	0.515
**TSH 0.37-2.49 mIU/L**	4310340 (73.80)	1.38 (0.85)	591546 (71.13)	1.46 (0.85)	<0.001
**TSH 2.50-4.87 mIU/L**	1160286 (19.86)	3.10 (0.86)	192938 (23.20)	3.12 (0.87)	<0.001
**TSH ≥4.88 mIU/L**	147087 (2.52)	5.94 (2.38)	28499 (3.43)	5.03 (2.49)	<0.001

Abbreviations: IQR, interquartile range; TSH, thyroid stimulating hormone.

[a] The Mann-Whitney U-test was used to examine the differences of TSH levels among two groups (Pregnant within six months *vs*. Pregnant after six months).

© 2021 Yang Y et al. *JAMA Network Open.*

eTable 2. Adjusted ORs of Adverse Pregnancy Outcomes According to Preconception TSH Levels

	Maternal preconception TSH (mIU/L) levels											
	TSH <0.10 mIU/L		TSH 0.10-0.36 mIU/L		TSH 0.37-2.49 mIU/L		TSH 2.50-4.87 mIU/L		TSH 4.88-9.99 mIU/L		TSH ≥10.00 mIU/L	
	(n = 59170)		(n = 164011)		(n = 4310340)		(n = 1160286)		(n = 125835)		(n = 21252)	
	n (%)	OR (95% CI)[a]	n (%)	OR (95% CI)	n (%)	OR	n (%)	OR (95% CI)	n (%)	OR (95% CI)	n (%)	OR (95% CI)
PTB	4589 (7.76)	1.23 (1.19-1.27)	11936 (7.28)	1.15 (1.13-1.18)	278457 (6.46)	1	77854 (6.71)	1.04 (1.03-1.05)	8982 (7.14)	1.13 (1.10-1.15)	1528 (7.19)	1.14 (1.08-1.20)
SGA	5840 (9.87)	1.37 (1.33-1.40)	13336 (8.13)	1.14 (1.12-1.17)	308367 (7.15)	1	82181 (7.08)	0.98 (0.97-0.99)	9556 (7.59)	1.05 (1.03-1.08)	1756 (8.26)	1.17 (1.11-1.23)
Birth defect	4 (0.01)	0.34 (0.13-0.91)	28 (0.02)	0.87 (0.57-1.27)	827 (0.02)	1	253 (0.02)	1.12 (0.97-1.29)	30 (0.02)	1.15 (0.79-1.66)	8 (0.04)	1.88 (0.93-3.78)
Perinatal infant death	240 (0.41)	1.26 (1.10-1.43)	599 (0.37)	1.14 (1.05-1.24)	13934 (0.32)	1	4041 (0.35)	1.08 (1.05-1.12)	530 (0.42)	1.31 (1.20-1.43)	100 (0.47)	1.47 (1.21-1.80)

Abbreviations: TSH, thyroid stimulating hormone; PTB, preterm birth; SGA, small for gestational age infant.

[a] ORs were adjusted by maternal age at last menstrual period, maternal education, area of residence, preconception body mass index, alcohol drinking, passive smoking, history of thyroid disease, hypertension, diabetes, history of adverse pregnancy outcomes.

© 2021 Yang Y et al. *JAMA Network Open.*

eTable 3. Analysis of Association Between Preconception TSH and Adverse Pregnancy Outcomes After Excluding Participants With Missing Data on Baseline Characteristics (N = 5 765 420)

	**Maternal preconception TSH (mIU/L) levels**											
	**TSH <0.10 mIU/L**		**TSH 0.10-0.36 mIU/L**		**TSH 0.37-2.49 mIU/L**		**TSH 2.50-4.87 mIU/L**		**TSH 4.88-9.99 mIU/L**		**TSH ≥10.00 mIU/L**	
	**(n = 58121)**		**(n = 161679)**		**(n = 4255397)**		**(n = 1145105)**		**(n = 124156)**		**(n = 20962)**	
	**n (%)**	**OR (95% CI)[a]**	**n (%)**	**OR (95% CI)**	**n (%)**	**OR**	**n (%)**	**OR (95% CI)**	**n (%)**	**OR (95% CI)**	**n (%)**	**OR (95% CI)**
PTB	4514 (7.76)	1.23 (1.19-1.27)	11766 (7.28)	1.15 (1.13-1.18)	274692 (6.46)	1	76782 (6.71)	1.04 (1.03-1.05)	8873 (7.15)	1.13 (1.10-1.15)	1507 (7.19)	1.14 (1.08-1.20)
SGA	5744 (9.88)	1.37 (1.33-1.40)	13125 (8.13)	1.14 (1.12-1.17)	304169 (7.15)	1	81031 (7.08)	0.98 (0.97-0.99)	9442 (7.60)	1.05 (1.03-1.08)	1734 (8.27)	1.17 (1.11-1.23)
Birth defect	4 (0.01)	0.34 (0.13-0.91)	27 (0.02)	0.87 (0.59-1.27)	817 (0.02)	1	250 (0.02)	1.11 (0.96-1.28)	29 (0.02)	1.15 (0.79-1.66)	8 (0.04)	1.88 (0.93-3.78)
Perinatal infant death	236 (0.41)	1.26 (1.11-1.43)	591 (0.37)	1.14 (1.05-1.24)	13770 (0.32)	1	3986 (0.35)	1.08 (1.05-1.12)	519 (0.42)	1.31 (1.20-1.44)	99 (0.47)	1.47 (1.21-1.80)

Abbreviations: TSH, thyroid stimulating hormone; PTB, preterm birth; SGA, small for gestational age infant.

[a] ORs were adjusted by maternal age at last menstrual period, maternal education, area of residence, preconception body mass index, alcohol drinking, passive smoking, history of thyroid disease, hypertension, diabetes, history of adverse pregnancy outcomes.

© 2021 Yang Y et al. *JAMA Network Open.*

eTable 4. Adjusted ORs of Adverse Pregnancy Outcomes According to Preconception TSH Levels Using 0.37-4.87 mIU/L of TSH as the Reference Group (N = 5 847 894)

	**Maternal preconception TSH (mIU/L) levels**									
	**TSH <0.10 mIU/L**		**TSH 0.10-0.36 mIU/L**		**TSH 0.37-4.87 mIU/L[Reference]**		**TSH 4.88-9.99 mIU/L**		**TSH ≥10.00 mIU/L**	
	**(n = 59170)**		**(n = 164011)**		**(n = 5470626)**		**(n = 125835)**		**(n = 21252)**	
	**n (%)**	**OR (95% CI)[a]**	**n (%)**	**OR (95% CI)**	**n (%)**	**OR**	**n (%)**	**OR (95% CI)**	**n (%)**	**OR (95% CI)**
PTB	4589 (7.76)	1.22 (1.18-1.26)	11936 (7.28)	1.14 (1.12-1.16)	356311 (6.51)	1	8982 (7.14)	1.12 (1.09-1.14)	1528 (7.19)	1.13 (1.07-1.19)
SGA	5840 (9.87)	1.37 (1.33-1.41)	13336 (8.13)	1.15 (1.13-1.17)	390548 (7.14)	1	9556 (7.59)	1.06 (1.04-1.08)	1756 (8.26)	1.18 (1.12-1.23)
Birth defect	4 (0.01)	0.33 (0.12-0.88)	28 (0.02)	0.84 (0.58-1.24)	1080 (0.02)	1	30 (0.02)	1.12 (0.77-1.62)	8 (0.04)	1.83 (0.91-

© 2021 Yang Y et al. *JAMA Network Open.*

														3.68)
Perinatal infant death		240 (0.41)	1.24 (1.09-1.41)		599 (0.37)	1.12 (1.03-1.22)		17975 (0.33)	1		530 (0.42)	1.29 (1.18-1.14)		100 (0.47)	1.45 (1.19-1.77)

Abbreviations: TSH, thyroid stimulating hormone; PTB, preterm birth; SGA, small for gestational age infant.

[a] ORs were adjusted by maternal age at last menstrual period, maternal education, area of residence, preconception body mass index, alcohol drinking, passive smoking, history of thyroid disease, hypertension, diabetes, history of adverse pregnancy outcomes.

© 2021 Yang Y et al. *JAMA Network Open.*
